# Correction: Spontaneous mutations and the origin and maintenance of quantitative genetic variation

**DOI:** 10.7554/eLife.22300

**Published:** 2016-10-19

**Authors:** Wen Huang, Richard F Lyman, Rachel A Lyman, Mary Anna Carbone, Susan T Harbison, Michael M Magwire, Trudy FC Mackay

Huang W, Lyman RF, Lyman RA, Carbone MA, Harbison ST, Magwire MM, Mackay TFC. 2016. Spontaneous mutations and the origin and maintenance of quantitative genetic variation. *eLife* **5**:e14625. doi: 10.7554/eLife.14625.

Published 23, May 2016

Due to an error in the computer code used to generate the table, gene symbols in supplemental file 1E were inadvertently shuffled, resulting in incorrect annotations of genes. The FlyBase IDs in this table were correct. This did not affect any analysis in the published paper, where only unique FlyBase IDs were used. The correct Supplementary file 1 with an updated table E is now provided. We apologize for the inconvenience.

## Additional files

Supplementary file 1. Supplemental tables. (A) Summary of DNA sequencing for MA lines. (B) List of detected mutations. (C) GO enrichment analysis of mutations. (D) Variance components of organismal traits among MA and DGRP lines. (E) Variance components of gene expression traits among MA and DGRP lines. (F) Comparison of mutational variance for genes within GO categories to that of genes outside the GO categories. (G) Comparison of apparent stabilizing selection for genes within within GO categories to that of genes outside the GO categories.

